# The cerebellar Golgi cell and spatiotemporal organization of granular layer activity

**DOI:** 10.3389/fncir.2013.00093

**Published:** 2013-05-17

**Authors:** Egidio D'Angelo, Sergio Solinas, Jonathan Mapelli, Daniela Gandolfi, Lisa Mapelli, Francesca Prestori

**Affiliations:** ^1^Department of Neuroscience, University of PaviaPavia, Italy; ^2^Brain Connectivity Center, IRCCS C. MondinoPavia, Italy; ^3^Department of Biomedical, Metabolic and Neural Sciences, University of Modena and Reggio EmiliaModena, Italy

**Keywords:** cerebellum, granular layer, Golgi cell

## Abstract

The cerebellar granular layer has been suggested to perform a complex spatiotemporal reconfiguration of incoming mossy fiber signals. Central to this role is the inhibitory action exerted by Golgi cells over granule cells: Golgi cells inhibit granule cells through both feedforward and feedback inhibitory loops and generate a broad lateral inhibition that extends beyond the afferent synaptic field. This characteristic connectivity has recently been investigated in great detail and been correlated with specific functional properties of these neurons. These include theta-frequency pacemaking, network entrainment into coherent oscillations and phase resetting. Important advances have also been made in terms of determining the membrane and synaptic properties of the neuron, and clarifying the mechanisms of activation by input bursts. Moreover, voltage sensitive dye imaging and multi-electrode array (MEA) recordings, combined with mathematical simulations based on realistic computational models, have improved our understanding of the impact of Golgi cell activity on granular layer circuit computations. These investigations have highlighted the critical role of Golgi cells in: generating dense clusters of granule cell activity organized in center-surround structures, implementing combinatorial operations on multiple mossy fiber inputs, regulating transmission gain, and cut-off frequency, controlling spike timing and burst transmission, and determining the sign, intensity and duration of long-term synaptic plasticity at the mossy fiber-granule cell relay. This review considers recent advances in the field, highlighting the functional implications of Golgi cells for granular layer network computation and indicating new challenges for cerebellar research.

## Introduction

The cerebellar Golgi cell was first identified through the pioneering investigations of C. Golgi (Golgi, 1874) and S. R. y Cajal (Ramón y Cajal, 1911), who predicted its function as a local interneuron. It was immediately clear from their studies that the Golgi cell was receiving a double excitatory input: from mossy fibers on the basal dendrites and from parallel fibers on the apical dendrites. Several decades later, other investigators demonstrated the inhibitory nature of Golgi cells (Eccles et al., 1964; Palay and Chan-Palay, 1974) and showed granular layer circuit organization to be based on characteristic double feedforward and feedback inhibitory loops directed toward granule cell dendrites in the cerebellar glomeruli (Eccles et al., 1966; Ito, 1984). The anatomical organization of these neurons also implied that Golgi cells generate a broad lateral inhibition extending beyond the afferent synaptic field. These discoveries suggested that the Golgi cell plays a central role in regulating granular layer activity (for an historical review of Golgi cell discovery, see Galliano et al., 2010) and, together with quantitative evaluation of cell numbers and convergence-divergence ratios in the cerebellar cortex, they became the basis of the classical models of cerebellar functioning (Marr, 1969; Albus, 1971; Ito, 1984). In recent years, advanced electrophysiological investigations have revealed important aspects of the molecular and cellular functions of these neurons. Most remarkably, Golgi cells have been shown to beat as theta-frequency pacemakers, to be entrained into coherent network oscillations, and to be efficiently activated by localized input bursts, which can phase-reset their activity. These properties were shown to exploit membrane mechanisms including specific ionic channels, excitatory, and inhibitory chemical synapses and dendritic gap junctions. Moreover, clarification of the function of the Golgi cell within the granular layer circuit demanded an extensive analysis at network level, which was carried out using voltage sensitive dye (VSD) imaging and multi-electrode array (MEA) recordings combined with mathematical simulations based on realistic computational models. These investigations highlighted the critical role of Golgi cells in: generating dense clusters of granule cell activity organized in center-surround structures, implementing combinatorial operations on multiple mossy fiber inputs, regulating transmission gain and cut-off frequency, controlling spike timing and burst transmission, and determining the sign, intensity and extension of long-term synaptic plasticity at the mossy fiber-granule cell relay. But unanswered questions remain. What is the exact nature of the relationship between these several and diverse activities and what is the exact role of Golgi cells in cerebellar computation?

## Fundamental properties of Golgi cells

Ever since their discovery (Golgi, 1874), Golgi cells have been the focus of considerable interest for both experimental and modeling studies (for previous updates see: Maex and De Schutter, 1998; De Schutter, 2000; Geurts et al., 2001; Maex and De Schutter, 2005; D'Angelo, 2008; Galliano et al., 2010). In recent years, new clues as to the functional properties of Golgi cells and their crucial role in the granular layer circuit have come from the field of cellular and synaptic physiology (Dieudonne, 1998; Forti et al., 2006; Solinas et al., 2007a,b, 2010; Vervaeke et al., 2010; Hull and Regehr, 2012). Golgi cells show a rich electrophysiological pattern and receive input, directly, and indirectly, from all kinds of fibers afferent to the cerebellar cortex and the circuits therein. We here revisit these findings and their implications.

### Fundamental properties of Golgi cells

**Figure 1:**
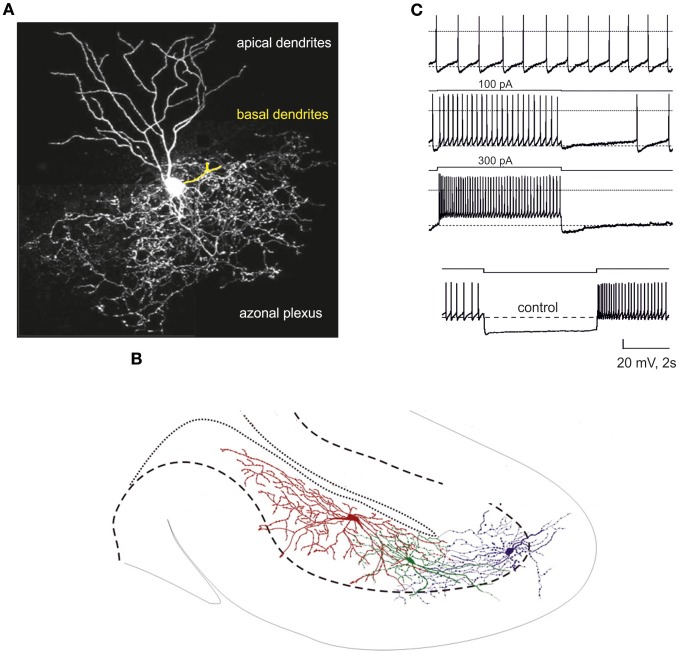
**The Golgi cell. (A)** Confocal microscopy reconstruction of a Golgi cell (adapted from http://www.regehr.med.harvard.edu). The Golgi cell has a large axonal plexus extending beyond the area covered by the dendrites. One of the basal dendrites has been colored in yellow. **(B)** Three main features of Golgi cell spatial organization: (i) the axons extend in the granular layer (delimited by a dashed line), (ii) the axons spread over the sagittal plane, (iii) the axonal fields of adjacent Golgi cells overlap (adapted from Barmack and Yakhnitsa, 2008). **(C)** Electroresponsiveness of a Golgi cell (adapted from Forti et al., 2006). The neuron shows low-frequency pacemaking and, upon depolarizing current injection, high-frequency spike discharge. Spike discharges are followed by an after-hyperpolarization and a silent pause. Upon hyperpolarizing current injection, the Golgi cell shows sagging inward rectification followed by a post-inhibitory rebound.

The fundamental anatomical properties of Golgi cells (Figure 1A) were first described in the histological studies of Golgi and Cajal (reviewed by Galliano et al., 2010). Golgi cells are the largest and most numerous interneurons of the granular layer (Golgi, 1874; Ramón y Cajal, 1888, 1995), which contains one Golgi cell to every several hundred or thousand granule cells (~6000 in cats Palkovits et al., 1971; ~1200 in humans: Andersen et al., 1992; ~400 in rats: Korbo et al., 1993). Typically, Golgi cells have an irregular soma (10–30 μm major diameter Dieudonne, 1998) giving off a series of basal dendrites, two or three apical dendrites and a widely ramified axon (Figure 1A Barmack and Yakhnitsa, 2008). Basal dendrites remain in the granular layer, while apical dendrites ascend into the molecular layer traversing the parallel fiber bundle. Golgi cells, although more abundant just below the Purkinje cell layer, can reside at different depths in the granular layer (Figure 1B). Attempts to identify Golgi cell subtypes by their biochemical fingerprints have revealed differential expression of certain biochemical markers (rat-303, calretinin, mGluR2, somatostatin, neurogranin) and of their coexpression with glycine, which can be co-released with GABA in certain Golgi cell subpopulations (Geurts et al., 2001, 2003; Simat et al., 2007). However, the absence of systematic differences in an extensive sample of electrophysiological recordings (Forti et al., 2006; Solinas et al., 2007a,b; Vervaeke et al., 2010; Hull and Regehr, 2012) (Figure 1C) suggests that biochemical differences between Golgi cells may not have an immediate impact on intrinsic electro responsiveness, but could regulate more subtle modalities of their activity.

### Golgi cell activation *in vivo*

**Figure 2:**
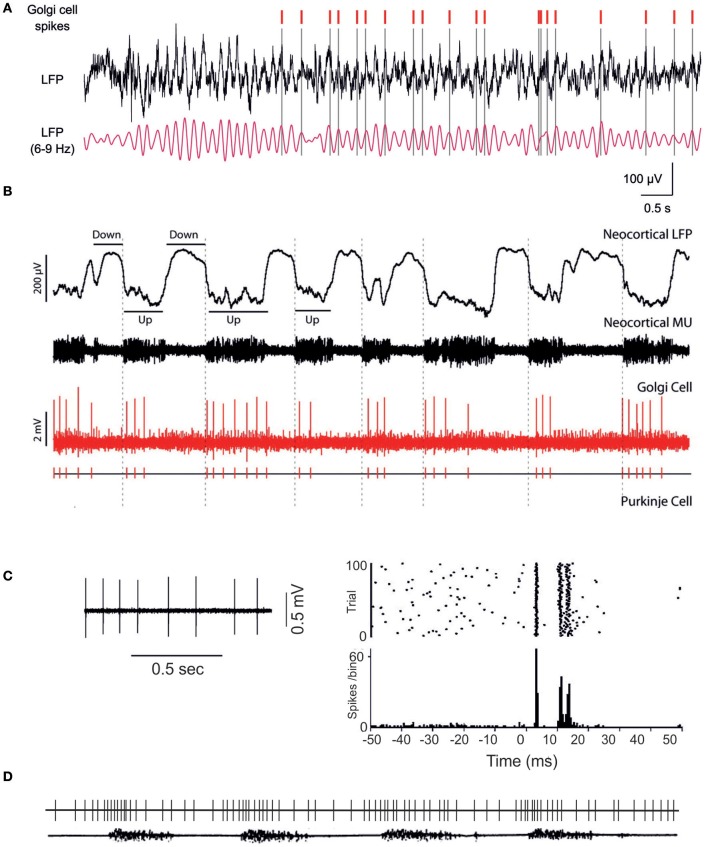
**Golgi cell activity *in vivo*. (A)** The Golgi cell spikes are in phase with oscillations in the local field potential of the granular layer (Dugue et al., 2009). **(B)** Golgi cells can show rhythmic entrainment with the UP-DOWN states characterizing neocortical activity (Ros et al., 2009). The different behavior in **(A)** and **(B)** may reflect different functional states or simply the fact that the trace in **(A)** may be part of an UP state as shown in **(B)**. **(C)** The Golgi cell shows background activity, over which punctate sensory stimulation elicits bursts of activity (from Holtzman et al., 2006). Each burst is usually composed of 2–3 spikes and is followed by a long-lasting inhibitory period (or silent pause in Vos et al., 1999a). **(D)** During locomotion, the Golgi cell is entrained into repetitive activity cycles, which modulate its discharge frequency (partially redrawn from Edgley and Lidierth, 1987). The lower trace shows the EMG of a limb muscle during walking.

Available information on the activity of Golgi cells *in vivo* is limited, but important (Figure 2). *In vivo*, Golgi cell firing is modulated by sensory inputs (Vos et al., 1999a; Holtzman et al., 2006; Barmack and Yakhnitsa, 2008; Xu and Edgley, 2008), sensorimotor activity (Edgley and Lidierth, 1987; van Kan et al., 1993; Prsa et al., 2009; Heine et al., 2010) and cortical UP/DOWN states (Ros et al., 2009). Punctate peripheral stimulation generates a short-latency excitation (Vos et al., 1999a; Holtzman et al., 2006; Xu and Edgley, 2008) comprising an early component attributed to direct inputs from mossy fibers and granule cells and a late component attributed to delayed inputs of cerebrocortical origin (Vos et al., 1999a). Convergence of parallel fiber excitation from multiple modules could explain the broad receptive fields of Golgi cells (Vos et al., 1999a; Holtzman et al., 2006; Xu and Edgley, 2008; Prsa et al., 2009; Heine et al., 2010; Holtzman and Jörntell, 2011), as well as Golgi cell firing synchronization along the parallel fiber bundle (Vos et al., 1999b). Thus, both feedback circuits and associative circuits may connect granule cells and Golgi cells in the cerebellar cortex. Interestingly, single Golgi cells can be entrained into oscillatory phases of cerebrocerebellar activity reflecting the UP/DOWN states of the cerebral cortex (Ros et al., 2009). It should also be noted that *in vivo* recordings have revealed effects that could be mediated by the climbing fibers, although the nature of the corresponding pathway remains uncertain (see below). These fundamental observations have also been explained on a cellular and connectivity basis.

## Cellular and synaptic properties of the Golgi cell revisited

Golgi cell activity and communication in the cerebellar network depend on the specific properties of the ionic and synaptic mechanisms involved.

### Membrane properties and intrinsic excitability

The electroresponsive properties of the Golgi cell remained unknown until recently, when intrinsic excitability was investigated in cerebellar slice preparations and subsequently modeled (Figure 1B Dieudonne, 1998; Forti et al., 2006; Solinas et al., 2007a,b). Golgi cells have a rich repertoire of electroresponsive properties, including pacemaking, resonance, phase-resetting and response patterns characterized by rebounds and response adaptations to depolarizing and hyperpolarizing inputs. Golgi cells in slices have been shown to beat regularly at around 6 Hz (Figure 1B Dieudonne, 1998; Forti et al., 2006; Solinas et al., 2007a,b) and to show increased spike frequency and precision when repetitively depolarized at this same frequency (Figure 1B Dieudonne, 1998; Forti et al., 2006; Solinas et al., 2007a,b). When hyperpolarized, they generate sagging inward-rectifying responses followed by a rebound bursts upon return toward the basal membrane potential level. When depolarized, they generate repetitive discharge characterized by spike-frequency adaptation and followed by a post-burst hyperpolarizing rebound upon return toward the basal membrane potential level. Interestingly, following a burst, Golgi cells phase-reset their own discharge, restarting pacing after a pause corresponding exactly to the oscillatory period. It should be noted that a recent paper did not report Golgi cell pacemaking *in vitro* (Dugue et al., 2009); the same paper reported weak adaptation during depolarizing steps, weak after-hyperpolarization (AHP) at the end of prolonged firing, and weak rebound after hyperpolarizing steps. These weak dynamic properties could reflect a specific functional state determined by strong electrical coupling with adjacent Golgi cells, which decreases the cell input resistance (see below). However, given the multiple effects of drugs used to test the effect of gap junctions [carbenoxolone interferes with voltage-dependent calcium channels, (Vessey et al., 2004), NMDA receptors (Tovar et al., 2009) and GABA receptors (Beaumont and Maccaferri, 2011)], doubts remain over the physiological implications of these findings. Using two-photon glutamate uncaging and dendritic patch-clamp recordings, it was recently shown that Golgi cells act as passive cables. They confer distance-dependent sublinear synaptic integration and weaken distal excitatory inputs. Gap junctions are present at a higher density on distal dendrites and contribute substantially to membrane conductance.

**Figure 3:**
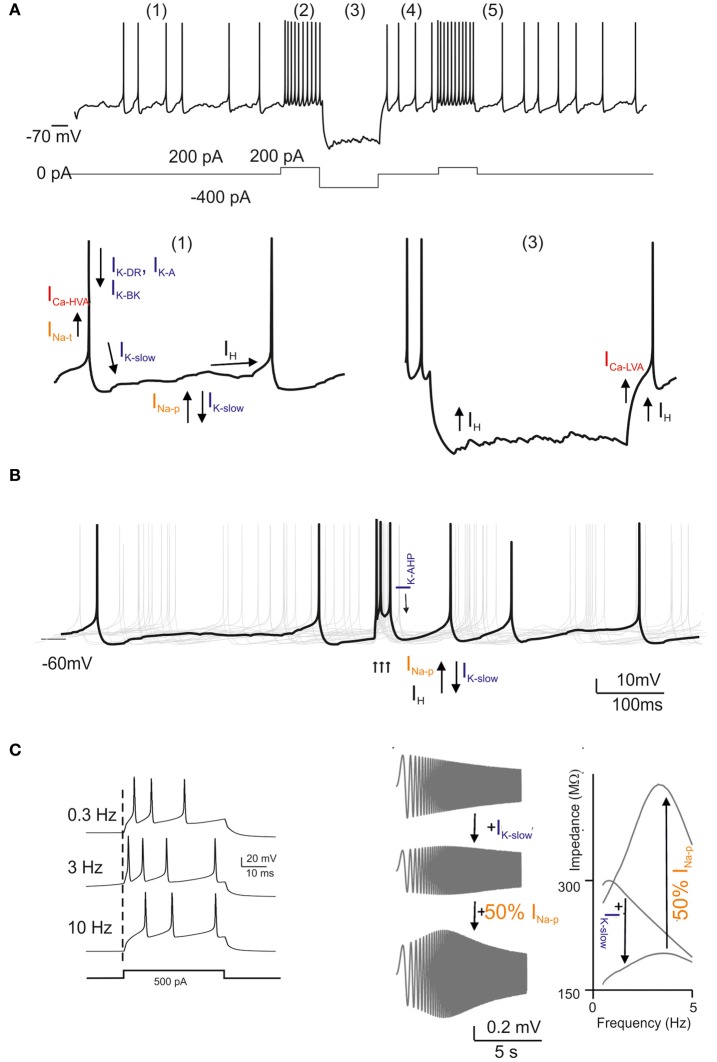
**Golgi cell ionic mechanisms.** This is a reconstruction of the ionic mechanisms of the Golgi cell membrane obtained using computational models (Solinas et al., 2007a,b) based on previous electrophysiological analysis (Forti et al., 2006) and incorporated into a large-scale granular layer model network (Solinas et al., 2010). Transient Na current (I_Na − t_); persistent Na current (I_Na − p_); resurgent Na current (I_Na − r_); high-voltage-activated Ca current (I_Ca − HVA_); Ca-dependent K current of the BK-type (I_K − BK_); Ca-dependent K current of the SK-type (I_K − AHP_); delayed-rectifier K current (I_KV_); slow K current of the M-type (I_K − slow_); fast-inactivating K current of the A-type (I_K − A_); slow inward-rectifier H-current (I_h_). In the different panels, the ionic channels involved are shown with arrows indicating their depolarizing or hyperpolarizing action. **(A)** Golgi cell responses like those reported in Figure 1A can be elicited by the model: (1) low-frequency pacemaking, (2) high-frequency spike discharge upon current injection, (3) sagging inward rectification, (4) post-inhibitory rebound, (5) phase resetting. **(B)** Golgi cell responses to bursts in the mossy fibers (arrows). After a burst, all responses are phase reset, generating an apparent “silent pause”. **(C)** Golgi cell responses to bursts in the mossy fibers repeated at different frequencies. Note that maximum responses are obtained around 6 Hz. The panels on the right show a “ZAP” protocol for investigating resonance. Resonance is determined by I_K − slow_ and is amplified by I_Na − p_.

The intrinsic electroresponsive properties of Golgi cells have been explained experimentally and subsequently modeled using a set of ionic channels (Figure 1B Dieudonne, 1998; Forti et al., 2006; Solinas et al., 2007a,b; see also Afshari et al., 2004) (Figure 3 Forti et al., 2006; Solinas et al., 2010). These are schematically reported below^1^:

1. Pacemaking depends on the action of four ionic currents, I_h_, I_Na − p_, I_K − AHP_, and I_K − slow_: I_h_ brings the membrane potential into the pacemaker region where the I_Na − p_/I_K − AHP_/I_K − slow_ interaction generates pacemaking.
2. Resonance is generated by I_K − slow_ and amplified by I_Na − p_.
3. Phase resetting is closely linked to calcium-dependent regulation of K currents. By being coupled to I_K − BK_, I_Ca − HVA_ enhances the fast phase of spike AHP, thereby resetting the spiking mechanism and sustaining high-frequency discharge.
4. Firing frequency regulation is based on the I_Na − f_/I_KV_ system and modulated by the I_K − BK_/I_Ca − HVA_ system.
5. Burst response following depolarization is enhanced by I_Na − r_ and delayed by I_K − A_; it is followed by spike frequency adaptation generated by the I_Ca − HVA_/I_K − AHP_ system and by I_K − slow_. Rebound excitation following hyperpolarization is generated by I_h_ and I_Ca − LVA_.
6. Dendritic integration and interneuronal network communication are enhanced by dendritic gap junctions.

Analysis of this pattern shows that different functionalities correspond directly to specific subsets of ionic channels. In particular, pacemaking and resonance both involve the I_Na − p_/I_K − slow_ system, and the pacemaker frequency is tuned by I_K − AHP_. Pacemaking requires I_h_, while phase resetting is based on the I_K − BK_/I_Ca − HVA_ system. A special role is played by the I_Na − f_/I_Na − p_/I_Na − r_ system, which controls various aspects of burst generation and resonance. Thus, although much remains to be done in terms of molecular characterization of the ionic channels involved, the available data are sufficient to allow precise modeling of the Golgi cell.

### Realistic modeling of Golgi cell activity

The realistic model of the Golgi cell (Solinas et al., 2007a,b) incorporates the mechanisms indicated above in the somatic compartment and maintains passive dendrites. This model, in turn incorporated into a detailed granular layer network model (Figure 3 Solinas et al., 2010), offers the following explanations for the main behaviors of the Golgi cell reported *in vivo*: pacemaking may underlie the rhythmic Golgi cell discharge *in vivo*, which, as a result of synaptic inputs, would then become irregular and spread over a broader frequency range (2–25 Hz); resonance could enhance Golgi cell entraining into coherent theta-frequency oscillations driven by cortical activity (see below), for example during sensorimotor behaviors like active whisking (Pellerin and Lamarre, 1997; Hartmann and Bower, 1998, 2001; Kleinfeld et al., 2006); the phase resetting of the pacemaker mechanism could provide the substrate of the “silent pause” observed after Golgi cell burst discharge (Vos et al., 1999a; Tahon et al., 2011); mechanisms enhancing spike bursting could determine the fast and precise Golgi cell responses to impulsive tactile stimuli (see also Morissette and Bower, 1996; Vos et al., 1999a, 2000; Volny-Luraghi et al., 2002; Tovar et al., 2009); firing frequency adaptation could help to limit Golgi cell spiking responses during prolonged stimulation (Tahon et al., 2011), and finally, the generation of rebounds in both the depolarizing and the hyperpolarizing directions could allow the Golgi cell to precisely follow the temporal evolution of afferent discharges observed during ongoing movement (Miles et al., 1980). Interestingly, by implementing the available realistic Golgi cell model (Solinas et al., 2007a,b) with dendritic gap junctions (Dugue et al., 2009; Vervaeke et al., 2010), it was shown that depolarization of one Golgi cell increased firing in its neighbors and enabled distal excitatory synapses to drive network activity more effectively. These mechanisms are tightly integrated with those governing chemical synaptic transmission, as explained below.

### Synaptic properties and circuit communication

The Golgi cell is extensively interconnected within the cerebellar network. In their classical analysis, which remains the fundamental reference for cerebellar circuit connectivity, Palay and Chan-Palay (1974) showed that Golgi cells receive a major excitatory input from mossy fibers, which form synapses on the basal dendrites, presumably in the glomeruli. Granule cells were reported to form their connections with Golgi cells through parallel fibers and also possibly through synapses *en passant* along the ascending axon. The climbing fibers have been suggested to form connections with Golgi cells, apparently by giving rise to thin collateral branches (called Scheibel's collaterals) just below the Purkinje cells which then reenter the upper part of the granular layer (Shinoda et al., 2000). Golgi cells have also been reported to receive inhibitory innervations from stellate/basket cells and Lugaro cells (Sotelo and Llinas, 1972). These original anatomical observations were corroborated by *in vivo* electrophysiological experiments, which showed that afferent activity, involving both the mossy fiber and the parallel fiber inputs, readily activated Golgi cells and that molecular layer interneurons could actually inhibit Golgi cells (Eccles et al., 1967).

**Figure 4:**
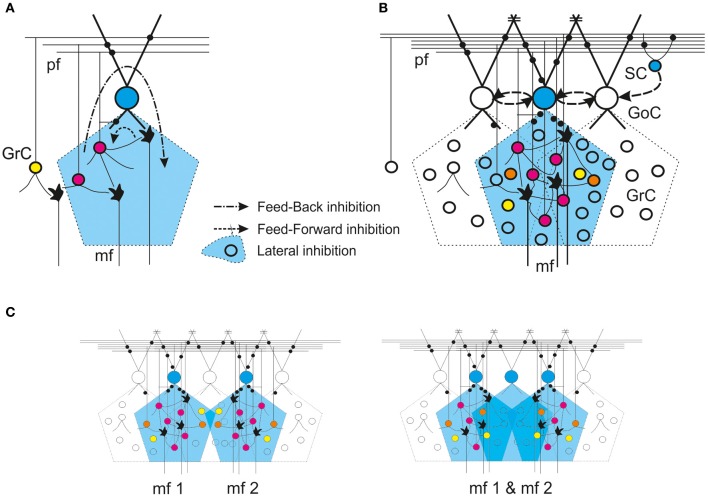
**Golgi cell connectivity.** Schematic representation of different modalities of Golgi cell connectivity. In all panels, the active Golgi cells are depicted in blue and the intensity of granule cell activity is scaled from blue (no activity) through yellow and orange to red (increasing activity). Synapses are in black. *GoC* Golgi cell, *GrC* granule cell, *SC* stellate cell, *mf* mossy fiber, *pf* parallel fiber. Inhibitory connections between Golgi cells and from stellate to Golgi cells are indicated with dashed arrow-lines. The gap junctions are indicated by =. **(A)** Feedforward and feedback loops. **(B)** Lateral inhibition. **(C)** Overlapping of axonal fields. Note that, in the area in common to two neighboring mossy fiber bundles, the circuit can generate *combined excitation* of the granule cells, but activation of an extra Golgi cell can generate a *combined inhibition*.

In the last decade, the concepts of synaptic connectivity have been refined through a combination of electrophysiological and morphological investigations, which have unveiled a complex organization of neurotransmitters and receptors. Moreover, forms of short-term and long-term synaptic plasticity (long-term depression, LTD, and long-term potentiation, LTP) and several modulatory effects have been reported, with the suggestion that these could provide the basis for regulating circuit dynamics, homeostasis and learning (for previous reviews see Geurts et al., 2003; Farrant and Nusser, 2005; D'Angelo, 2008). Recent advances have increased our understanding of this complex system and allowed us to re-design the picture of the loops involved (Figure 4), although some controversies remain.

### Excitatory synapses with Golgi cells

The main excitatory inputs to Golgi cells are glutamatergic. Recent studies report the involvement of AMPA (Kanichay and Silver, 2008) and NMDA receptors (Cesana et al., 2010) at mossy fiber-Golgi cell relays. These synapses show moderate short-term depression; this makes the Golgi cells highly sensitive to mossy fiber afferent bursts, so that the Golgi cells then elicit new bursts in response to the input. Instead, activation of AMPA, NMDA and kainate receptors has been reported at parallel fiber-Golgi cell relays (Dieudonne, 1998; Bureau et al., 2000; Misra et al., 2000). While AMPA receptor-mediated currents undergo a marked short-term depression, kainate receptor responses are summed, enhancing temporal summation during repetitive parallel fiber activity. Thus, this synapse may be able to transmit both temporally precise single granule cell spikes and granule cell bursts (Chadderton et al., 2004; Rancz et al., 2007). Recently, a form of LTD was reported following intense high frequency stimulation of parallel fibers (Robberechts et al., 2010), although it remains to be established whether or not this LTD exists in the presence of natural patterns of stimulation. Metabotropic glutamate receptors also appear to regulate Golgi cell circuit functions. The mGluR2 receptors are expressed in Golgi cells (Geurts et al., 2001) and their activation enhances an inward rectifier K current which helps to silence the Golgi cell following intense granule cell-Golgi cell transmission (Watanabe and Nakanishi, 2003). This mGluR2-dependent mechanism may facilitate the transmission of protracted bursts along the mossy fiber-granule cell pathway (Arenz et al., 2008).

### Inhibitory synapses with Golgi cells

The inhibitory inputs to Golgi cells are GABAergic or glycinergic. Pure GABAergic inputs have been suggested to come from stellate and basket cells, and mixed GABAergic glycinergic inputs from Lugaro cells (Dumoulin et al., 2001). The glycinergic inhibitory postsynaptic current (IPSC) component, being expressed in variable amounts and having the capacity to slow down IPSC kinetics, can fine tune the duration of Golgi cell inhibition (Dumoulin et al., 2001). Evidence was recently provided indicating that GABAergic Golgi cells are inhibited by other Golgi cells rather than by molecular layer interneurons (Hull and Regehr, 2012). This report is somewhat controversial, however, in that *in vivo* electrophysiological recordings have clearly shown Golgi cell inhibition to be the consequence of a disynaptic pathway passing through granule cells and molecular layer interneurons (Eccles et al., 1967). The reason for this discrepancy remains to be determined.

To our knowledge, molecular layer interneurons remain the best candidates to mediate feedback inhibition deriving from activity in parallel fibers (and climbing fibers, see below) toward Golgi cells. Golgi cell inhibition by molecular layer interneurons could enhance post-inhibitory rebounds in granule cell activity and could also explain the long-lasting depressions of firing induced by strong electrical stimuli (Holtzman et al., 2006). Golgi cell inhibition indirectly caused by climbing fibers and molecular layer interneurons could also have the important effect of synchronizing the granular layer with the inferior olive, the molecular layer and the deep cerebellar nuclear circuits. Conversely, Golgi cell-Golgi cell synapses could serve to dampen and equalize Golgi cell responses within the granular layer circuit. While it is possible that the two mechanisms coexist, their relative importance in different functional conditions remains to be determined. Finally, inhibition coming from Lugaro cells has been reported to depend on serotonergic activation of these neurons (Dieudonne and Dumoulin, 2000). This would allow Lugaro cells to correlate Golgi cell activity with general functional states of the brain.

### Indirect inhibitory effect of climbing fibers on Golgi cells

Although climbing fibers are glutamatergic, there exists electrophysiological evidence that they have an inhibitory effect on Golgi cells. *In vivo* recordings, synchronous stimulation of climbing fibers and peripheral afferents elicited a long-lasting depression of the Golgi cell inhibitory input to granule cells (Xu and Edgley, 2008), although the underlying mechanism remains unclear. Indeed, despite evidence of climbing fiber ramifications in the proximity of Golgi cells, i.e., the aforementioned Scheibel's collaterals (Shinoda et al., 2000), the presence of effective synaptic connections between climbing fibers and Golgi cells remains uncertain. A recent study that used advanced immunohistochemical techniques and 3D reconstruction, while supporting the prominent apposition of climbing fibers to Purkinje cells and molecular layer interneurons, did not provide comparable evidence for Golgi cells, thus arguing against a functional significance of direct synaptic contacts between climbing fibers and Golgi cells (Galliano et al., 2013). This negative result does not exclude the possibility that spillover of glutamate from climbing fibers in the proximity of Golgi cell dendrites could activate mGluR2 receptors, thereby causing a long-lasting modulation of the Golgi cell response. Another possibility is that glutamate spillover from climbing fibers causes plastic changes at parallel fiber-molecular layer interneuron synapses (Mathews et al., 2012). These mechanisms remain to be investigated.

### Golgi cell-Golgi cell communication through dendritic gap junctions

Reported in early studies, the finding of gap junctions in Golgi cell dendrites suggested that these neurons could be electrically coupled with each other and with molecular layer interneurons (Sotelo and Llinas, 1972). Recently, functional evidence for gap junctions connecting Golgi cell apical dendrites was reported (Dugue et al., 2009; Vervaeke et al., 2010). This interconnection endows Golgi cells with a further level of complexity. Golgi cells are known to loosely synchronize their activity (Vos et al., 1999b) and this effect could be explained by their shared parallel fiber input (Maex and De Schutter, 1998). The gap junctions provide a further electrical link between Golgi cells which is capable of accelerating the rise and enhancing the stabilization of synchronous oscillations. Moreover, counter-intuitively, the heterogeneity of the conductance of the electrical connections gives rise to a transient desynchronization of adjacent Golgi cells driven by external stimuli (Vervaeke et al., 2010). The real relevance of gap junctions and their relative contribution to overall activity states of the cerebellar cortex *in vivo* remains largely to be determined.

### Inhibition of granule cells by Golgi cells

The synaptic output of Golgi cells is GABAergic and it inhibits the granule cells in the cerebellar glomeruli. The IPSCs consist of a fast and a slow component (Rossi et al., 2003) determined by differential receptor subtypes (Farrant and Nusser, 2005). The α1 subunit-containing receptors are localized in the synaptic cleft and are mainly involved in bringing about the IPSC peak. The α6 subunit-containing receptors, which have a high affinity for GABA, a low desensitization rate and are distributed from the synaptic junction to destinations several hundreds of nanometers apart, help to enhance the IPSC tail through a spillover-dependent mechanism (Tia et al., 1996; Nusser et al., 1998; Rossi and Hamann, 1998; Brickley et al., 1999; Hadley and Amin, 2007). This double receptor system is probably important for ensuring extremely precise timing of inhibition onset and, at the same time, efficient temporal summation during trains of Golgi cell spikes. As well as causing phasic inhibition, Golgi cells can contribute to the regulation of basal granule cell input conductance by helping to maintain a tonic GABA concentration level inside the glomerulus (Brickley et al., 1996; Chadderton et al., 2004; Duguid et al., 2012). This tonic level of GABA is thought to activate high-affinity receptors (Tia et al., 1996) primarily, but also to control the gain of the mossy fiber-granule cell relay (Mitchell and Silver, 2003) (see below).

In contrast to the inhibitory action exerted by fast GABAergic synaptic transmission and tonic inhibition, some other mechanisms limit the impact of Golgi cell inhibition in a homeostatic manner. A transient feedback depression of neurotransmitter release probability is determined by ambient GABA through presynaptic GABA-B autoreceptors and limits the first response in a burst (Mapelli et al., 2009). Two forms of medium-term adaptation of granule cell responses have been reported to occur through activation of postsynaptic GABA-B receptors. Both application of the GABA-B receptor agonist, baclofen, and spike bursts in Golgi cell axons can induce depression of the inward rectifier K current in granule cells causing membrane depolarization and (Rossi et al., 2006) depression of the GABA-A receptor-mediated current in granule cells reducing the inhibitory effect (Brandalise et al., 2012). These mechanisms, as well as glomerular crosstalk (Mitchell and Silver, 2000b, see below), could have an homeostatic effects and be responsible for the protracted granule cell responses to mossy fiber bursts observed in VSD recordings (Mapelli et al., 2010a).

### Glomerular functions: spillover, crosstalk and tonic inhibition

The control of granule cell activity by Golgi cells occurs almost exclusively in the glomerulus, which is a specialized structure in which the ambient concentration of neurotransmitters can be effectively regulated. The glomerular compartment, enwrapped in a glial sheet, is thought to act as a diffusion barrier entrapping neurotransmitter molecules, enhancing the effects of spillover, and giving rise to tonic inhibition (Barbour and Häusser, 1997; Rossi and Hamann, 1998; Hamann et al., 2002). Moreover, the close apposition of presynaptic and postsynaptic elements of both excitatory and inhibitory fibers enhances processes of synaptic crosstalk.

In the glomerulus, a tonic GABA level is established and regulated by the rate of vesicular release from Golgi terminals and the rate of non-vesicular release and re-uptake in glial cells (Rossi et al., 2003). Recently, the tonic component of granule cell inhibition was shown to depend largely on the GABA released by glial cells through bestrophin-1 anion channels (Lee et al., 2010). The contribution of non-vesicular GABA release from Golgi cells may be increased by acetylcholine (Rossi et al., 2003, see below).

There exists functional evidence of crosstalk between mossy fiber and Golgi cell terminals due to neurotransmitter spillover in the glomerulus, which results in heterosynaptic activation of presynaptic GABA and glutamate autoreceptors. Glutamate spillover from mossy fiber terminals on granule cells activates presynaptic mGluR2 receptors on Golgi cell terminals and inhibits GABA release (Mitchell and Silver, 2000a), while GABA spillover from Golgi cell-to-granule cell synapses activates presynaptic GABA-B receptors on mossy fiber terminals and inhibits glutamate release in a frequency-dependent manner (Mitchell and Silver, 2000b). These reciprocal actions may reinforce the switch from excitation to inhibition of granule cells, in such a way that once excitation prevails it becomes even more dominant over inhibition (and vice versa when inhibition prevails).

The combination of crosstalk and tonic inhibition orchestrates a complex control of the granule cell input/output relationship. Tonic inhibition leads to a reduction of granule cell excitability, so that the slope of the input/output curve does not change and the frequency of the emitted spikes is similarly reduced at all input intensities (Brickley et al., 1996; Chadderton et al., 2004; Duguid et al., 2012). Conversely, crosstalk changes the slope of the firing input/output curve in a more complex manner, dampening responses during high-frequency mossy fiber-granule cell transmission and thus altering granule cell sensitivity to changes in input frequency (Mitchell and Silver, 2003). Finally, a crosstalk effect could also be mediated by glycine, which is co-released with GABA by Golgi cells (Dugué et al., 2005). Granule cells do not express glycine receptors, but it is tempting to speculate that glycine plays a role in regulating activation of granule cell NMDA receptors on their glycine binding site (D'Angelo et al., 1990). Conversely, both GABA and glycine receptors are expressed in unipolar brush cells (UBCs), in which Golgi cell activity generates mixed GABAergic/glycinergic responses (Dugué et al., 2005).

## Functional connectivity: expanding the view

Golgi cell connectivity is closely bound up with the organization of the entire granular layer circuit (Eccles et al., 1967; Palay and Chan-Palay, 1974). At microscopic level, statistical rules govern the way granule cells, Golgi cells and mossy fibers are wired together to form local networks. On an intermediate scale, two network-organizing principles are especially relevant: the formation of “granule cell clusters” and of “center-surround” structures. The clusters, revealed by measuring or imaging the area activated by sensory punctate stimuli, are formed by 200–600 adjacent granule cells (Roggeri et al., 2008; Diwakar et al., 2011). In these clusters, the excitatory-inhibitory (E/I) balance is higher in the core, thus leading to the formation of a center-surround structure (Mapelli and D'Angelo, 2007). It is also important to consider how Golgi cell connectivity is related to the modular organization of the cerebellar cortex [(Voogd et al., 2003; Apps and Hawkes, 2009; Oberdick and Sillitoe, 2011); for a recent review see (D'Angelo and Casali, 2012)]^2^. At this level, the granular layer performs complex operations of spatiotemporal recoding of the mossy fiber input, which can be recognized by analyzing intermodular connectivity and signal transmission along the vertical and transverse axis of the cerebellar cortex (Bower and Woolston, 1983; Mapelli et al., 2010a,b).

### Synaptic organization in the granular layer

Ultrastructural measurements have revealed that each granule cell receives, on average, three Golgi cell inhibitory synapses on as many different dendrites (Hamori and Somogyi, 1983; Jakab and Hamori, 1988). Golgi cell-granule cell synapses consist of small boutons located proximally to granule cell dendritic endings, which, in turn, receive excitatory mossy fiber terminals. Both mossy fiber and Golgi cell terminals, together with several tens of granule cell dendrites (Palkovits et al., 1971; Hamori and Somogyi, 1983) and Golgi cell basal dendrites are included in the cerebellar glomerulus. These investigations have opened up several physiological issues.

For example, there is the question of whether Golgi cell synapses impinging on a granule cell originate from the same or from different Golgi cells. Typically, multiple IPSCs in a granule cell can be recruited by increasing the stimulation intensity (Mapelli et al., 2009), which is consistent with 3–5 independent Golgi cells connected. Indeed, the frequency of spontaneous IPSCs, which are synaptic events determined by intrinsic activity in Golgi cells, exceeds the pacemaker frequency shown by single Golgi cells, further supporting the convergence of multiple Golgi cells onto the same granule cells.

Given that glomeruli receive an average of 53 dendrites (an estimate obtained from rat cerebellum: Jakab and Hamori, 1988) from as many different granule cells, another issue is whether a Golgi cell innervates all the granule cells impinging on the same glomerulus. Even a minimal stimulation (i.e., that activates a single synaptic contact) can elicit a direct and an indirect spillover-mediated component in granule cell IPSCs (Mapelli et al., 2009). Since spillover is a sign of release on neighboring synapses in the glomerulus (Rossi and Hamann, 1998), this indicates that a Golgi cell axon forms more than one synapse inside a glomerulus and inhibits numerous (if not all) granule cell dendrites in that glomerulus.

Therefore, in theory, a single Golgi cell should not innervate a granule cell more than once (and therefore should not innervate other glomeruli within reach of the dendrites of proximal granule cells) and each glomerulus should be innervated by a single Golgi cell. This configuration favors the expansion of the Golgi cell axonal field and the integration of multiple Golgi cell axons within the same volume of the granular layer (Solinas et al., 2010). Finally, it has been shown that, in the vestibulo-cerebellum, in addition to granule cells, Golgi cells also inhibit UBCs (Dugué et al., 2005).

### Quantitative Golgi cell connection scheme

On the basis of current knowledge it is possible to generate a quantitative connection scheme for the Golgi cell, which is unique both for its high level of precision and for the quantity of available experimental data. Using morphological measurements, it can be calculated that the rat cerebellar granular layer has a cell density of 4 × 10^6^/mm^3^ for granule cells and 9300/mm^3^ for Golgi cells, with a Golgi cell:granule cell ratio of 1:430 (Korbo et al., 1993). Moreover, the density of the glomeruli is 3 × 10^5^/mm^3^, and each glomerulus is composed of one mossy fiber terminal, about 53 dendrites from separate granule cells (Jakab and Hamori, 1988), and one or more dendrites from Golgi cells. Network connections can be reconstructed by applying simple rules, most of which can be directly extracted from original works on cerebellar architecture (e.g., see Eccles et al., 1967).

Granule cell connection rules are quite simple and can be summarized as follows: granule cell dendrites cannot reach glomeruli located more than 40 μm away (mean dendritic length: 13.6 μm) and a single granule cell cannot send more than one dendrite into the same glomerulus. Conversely, Golgi cell connection rules are more complex. It can be assumed that only one Golgi cell axon enters a glomerulus, forming inhibitory synapses on all the afferent granule cell dendrites, and that a Golgi cell axon entering a glomerulus cannot access the neighboring glomeruli if they share granule cells with the first one. This should prevent a granule cell from being inhibited twice by the same Golgi cell (see above and Solinas et al., 2010). Each Golgi cell can inhibit as many as 40 different glomeruli and a total of about 2000 granule cells, accounting for the 1:430 Golgi cell:granule cell ratio and the aforementioned convergence and divergence ratios (see above). Recent calculations seem to indicate a specific organization of excitatory connectivity. Golgi cells were suggested to receive excitatory inputs from about 40 mossy fibers on basal dendrites (Kanichay and Silver, 2008). Moreover, a specific organization is emerging for granule cell inputs through the ascending axons and parallel fibers (Cesana et al., 2010). Golgi cells could receive about 400 connections from the ascending axons of local granule cells on the basal dendrites and another 400 connections through the parallel fibers of local granule cells, which would provide the basis for a powerful feedback circuit. In addition, Golgi cells receive about 1200 parallel fiber contacts on the apical dendrites from transversely organized granular layer fields. It has been calculated that the effectiveness of local granule cells is about 10 times greater than that of an equivalent population located outside the direct afferent field and forming only parallel fiber contacts toward the Golgi cell.

Much less is known about inhibitory connections. Each Golgi cell receives inhibitory input from several dozen molecular layer inhibitory interneurons (stellate cells and Basket cells) (Dumoulin et al., 2001) and from other Golgi cells (Hull and Regehr, 2012). Moreover Golgi cell dendrites are coupled through gap junctions (Vervaeke et al., 2010) and may also be connected with molecular layer interneurons in the same way (Sotelo and Llinas, 1972).

### Modular and intermodular connectivity of Golgi cells

There are several other anatomical aspects that help to shed light on Golgi cell wiring and account for the most important aspects of Golgi cell functions in the cerebellar cortex. First, the Golgi cell axonal plexus extends exclusively in the granular layer and, through thin branches, can form secondary plexuses in the same or even in neighboring laminae (Figure 1A, see Eccles et al., 1967; Barmack and Yakhnitsa, 2008). The broader extension of the axonal plexus compared to the basal dendrites provides the basis for lateral inhibition and for intermodular connectivity. Second, axonal plexuses coming from different Golgi cells overlap (Figure 1A, see Barmack and Yakhnitsa, 2008). This property, by causing the convergence on one granule cell of inhibition from more than one Golgi cell, is necessary to allow the combinatorial inhibition of granule cells. Third, as revealed by immunostaining for zebrin-2, aldolase C and other markers (Sillitoe et al., 2008), Golgi cells emit their apical dendrites within Purkinje cell compartments. Fourth, the Golgi cell axonal field extends along the sagittal plane (Figure 1A Barmack and Yakhnitsa, 2008), as do mossy fiber (Wu et al., 1999; Sultan and Heck, 2003) and climbing fiber branching (Shinoda et al., 2000). Therefore, Golgi cell wiring appears rather complex: through mossy fiber inputs to their dendrites, Golgi cells are preferentially wired within microcircuits involving anatomically organized olivo-cerebellar and mossy fiber compartments (Brown and Bower, 2001, 2002; Voogd et al., 2003; Pijpers et al., 2006); meanwhile, through their apical and basal dendrites and axonal plexus (mean mediolateral extent is 180 ± 40 μm equivalent to 10–15 PC dendrites Barmack and Yakhnitsa, 2008), they are interconnected with multiple such compartments. Fourth, unlike Purkinje cell dendrites, Golgi cell dendrites are not rigorously organized on a plane but rather in a three-dimensional structure. Thus, Golgi cells may not be equipped to detect ordered temporal sequences transmitted through parallel fibers (Braitenberg, 1967; Braitenberg et al., 1997). This topographical organization is further complicated by the fact that parallel fibers cross several Golgi cell dendritic arbors along the transverse axis, while mossy fibers ramify along the sagittal axis (Wu et al., 1999; Sultan and Heck, 2003). Therefore, Golgi cell inhibition can be redistributed to mossy fiber clusters in the parasagittal plane.

Interestingly, granule cells have recently been reported to show a high rate of connectivity with local Golgi cells, and thus to implement a powerful feedback in the local microcircuit (Cesana et al., 2010). This, together with lateral inhibition, is probably one of the factors underlying the granule cell cluster organization shown on *in vivo* recordings (Diwakar et al., 2011) and the center-surround organization revealed in network imaging experiments (Mapelli and D'Angelo, 2007; Mapelli et al., 2010a,b). These observations, combined with electrophysiological and modeling data, support the view that Golgi cells can respond precisely to topographically organized inputs, but also perform widespread spatiotemporal integration of parallel fiber information (Vos et al., 1999a,b, see also below; De Schutter and Bjaalie, 2001).

### Modeling Golgi cell interactions in the granular layer

The cerebellar cortical network, characterized by a beautiful and regular connection matrix with rectangular symmetry (Braitenberg, 1967), stimulated the development of a series of network models (Pellionisz and Szentagothai, 1973; Pellionisz and Llinás, 1979; Buonomano and Mauk, 1994; Maex and De Schutter, 1998, 2005; Medina and Mauk, 2000; Santamaria et al., 2007). However, the recent advances in understanding of Golgi cell connectivity could significantly change our view on the role these neurons play in shaping the dynamics of the granular layer. A first attempt at reconstructing the complex connectivity, the intrinsic excitability and the short-term synaptic dynamics of the granular layer cell was made by Solinas et al. (2010), and further developed Solinas and D'Angelo (2012), who considered a homogeneous portion of the granular layer including about 4000 granule cells and a proportionate number of Golgi cells and glomeruli and accounted for the topological constraints relevant on this relatively limited scale. Relaxing these topological constraints and transforming the connections from an organized into a random mesh (thus respecting only the numerical proportions of the elements) reduced the spatial discrimination and temporal precision of the responses to incoming mossy fiber inputs. Thus, the specific topology could indeed have a relevant functional significance in terms of spatial pattern separation and elaboration of internal temporal circuit dynamics. An explicit representation of the glomerulus with internal diffusion allowing for independent generation of direct and indirect IPSCs may further improve this description. Further modeling and simulations are needed to clarify the impact of the connectivity properties of Golgi cells on a larger scale and thus to account for their intermodular organization and for the 3D organization of the connections. In particular, it seems important to incorporate the effect of inhibition among Golgi cells, the effect of gap junctions between Golgi cells, the local connectivity rules determining appropriate proportions of excitatory synapses made by local granule cell patches along the ascending axon and parallel fibers, and the clustering of mossy fiber rosettes in the parasagittal plane. This could provide further insight into the importance of the topological organization of Golgi cells.

The resetting of Golgi cell activity was also studied using this Golgi cell model in a recent simulation study that showed the impact of dendritic gap junctions on the activity of sets of Golgi cells and their desynchronization driven by external stimuli (Vervaeke et al., 2010, all these models are available at http://senselab.med.yale.edu/ModelDB).

## Coherence and individuality of Golgi cell activity

A fundamental functional property of neuronal networks is that of sustaining *coherent oscillations*: this occurs in such a way that neurons helping to generate the oscillations are, at the same time, themselves entrained into the oscillation. Akin with this, *resonance is* a condition occurring when a physical system undergoes a periodic activation with a frequency equal or similar to the intrinsic oscillation frequency of the system itself, so that such a system tends to oscillate at its maximum amplitude (French, 1971) (Figures 2, 3). The counterpart of neuronal coherence is neuronal individuality. After all, neuronal networks would not be of much use if they had to beat with all their elements always fully synchronized. The mechanism that allows neurons to temporarily escape the coherent circuit pulsation is *phase resetting* (Llinas, 1988; Buzsaki, 2006) (Figures 2, 3). Interestingly, the Golgi cell expresses specific ionic channels and mechanisms that allow an independent control over oscillation, resonance and phase resetting (Solinas et al., 2010, see above).

### Coherent oscillations and resonance

The Golgi cell has been suggested to play a critical role in generating granular layer circuit oscillations and resonance. An important observation in this respect is that the theta band has specific relevance for brain functioning and important implications for the cerebellum (D'Angelo et al., 2009). Theta-frequency oscillations are observed in the granular layer during resting activity in the awake rat and monkey (Pellerin and Lamarre, 1997; Hartmann and Bower, 1998; Courtemanche et al., 2009) and theta rhythms are observed using magneteoencephalography in awake humans (Kujala et al., 2007). Computational analysis indicates that these oscillations require an intact feedback inhibitory loop and generate a loose synchrony of the neurons involved (Maex and De Schutter, 1998; Solinas et al., 2010). Interestingly, granular layer theta-frequency oscillations are correlated with cerebrocortical activity (O'Connor et al., 2002; Gross et al., 2005; Ros et al., 2009). Golgi cells, being theta-band pacemakers, could contribute to the maintenance of cerebrocerebellar coherence, and being integrated into a syncytium, could also help to maintain granular layer coherence. An evolution of this concept is the finding that the rhythmic activity of Golgi cells can be tuned, depending on the strength of gap junction connectivity, within a few and 20 Hz (Dugue et al., 2009). Therefore, the Golgi cell interneuron network may retune itself and regulate the sensitivity toward cerebrocortical activity over a broad frequency band. Factors controlling the strength of Golgi cell electrical coupling remain to be identified.

As the concept of oscillation is akin to that of resonance, what is the relationship between resonance and oscillations in the granular layer? The nature of both phenomena depends on the physical particularities of the system involved. We recently observed that granular layer resonance reflects intrinsic properties of granule cells (D'Angelo et al., 2001; Gandolfi et al., 2013), while resonance and oscillations in the inhibitory interneuron network require gap junctions between Golgi cells (Dugue et al., 2009) and possibly also intrinsic pacemaking in Golgi cells (Forti et al., 2006). Thus, the granular layer circuit appears to be composed of multiple resonators (the mossy fiber-granule cell synapse and the Golgi cell inhibitory network) coupled one to the other and tuned within the same frequency range. The Golgi cells also provide synchronicity through lateral inhibition and can enhance resonance in the granule cell population (Gandolfi et al., 2013). In aggregate, resonance can amplify the granule cell output when the mossy fiber input is conveyed at theta frequency. At this frequency the inhibitory circuit can spontaneously oscillate, thereby creating a condition in which the system is able to optimize phase locking, information transmission and, potentially, the induction of long-term synaptic plasticity.

### Cell individuality and phase resetting

Golgi cells show a high sensitivity to sensory inputs, responding in about 10 ms to punctate sensory stimulation (Vos et al., 1999a; Kanichay and Silver, 2008; Xu and Edgley, 2008; Tahon et al., 2011). This response mechanism, based on specific ionic channels distinct from those causing oscillations and resonance (see above), allows Golgi neurons to be phase reset. Thus, after a local stimulus, specific Golgi cells could escape the coherent theta cycle entraining the inhibitory network and generate a specific regulation of spike transmission through those granule cells that are under their inhibitory control. Indeed, a loose rather than a tight coherence among Golgi neurons has been reported, possibly indicating that although these neurons tend to be paced with each other, at the same time a few of them can escape the coherent oscillation to generate a specific signal in a meaningful phase relationship with the diffuse theta oscillation (Vos et al., 1999b; Volny-Luraghi et al., 2002). Extensive phase resetting may contribute to the desynchronization of granular layer local field potential oscillations when the rat is passing from resting attentiveness to active motor behavior (Hartmann and Bower, 1998).

Just as feedback inhibition is critical in bringing about oscillations in the Golgi interneuron network, feedforward inhibition is critical in bringing about phase resetting. Network simulations have shown that the balance of the two mechanisms is also critical, oscillations being prevented when the feedforward loop is strong compared with the feedback loop (Maex and De Schutter, 1998; Solinas et al., 2010). The balance of the two loops *in vivo* remains an open issue. It is tempting to speculate that diffuse activity in the parallel fibers might sustain coherent oscillations in large Golgi cell populations, while a sufficiently strong input in a mossy fiber subset could phase reset Golgi cells, thus allowing local control of transmission through dedicated granular layer channels.

## Control of signal transmission and plasticity at the mossy fiber-granule cell relay

**Figure 5:**
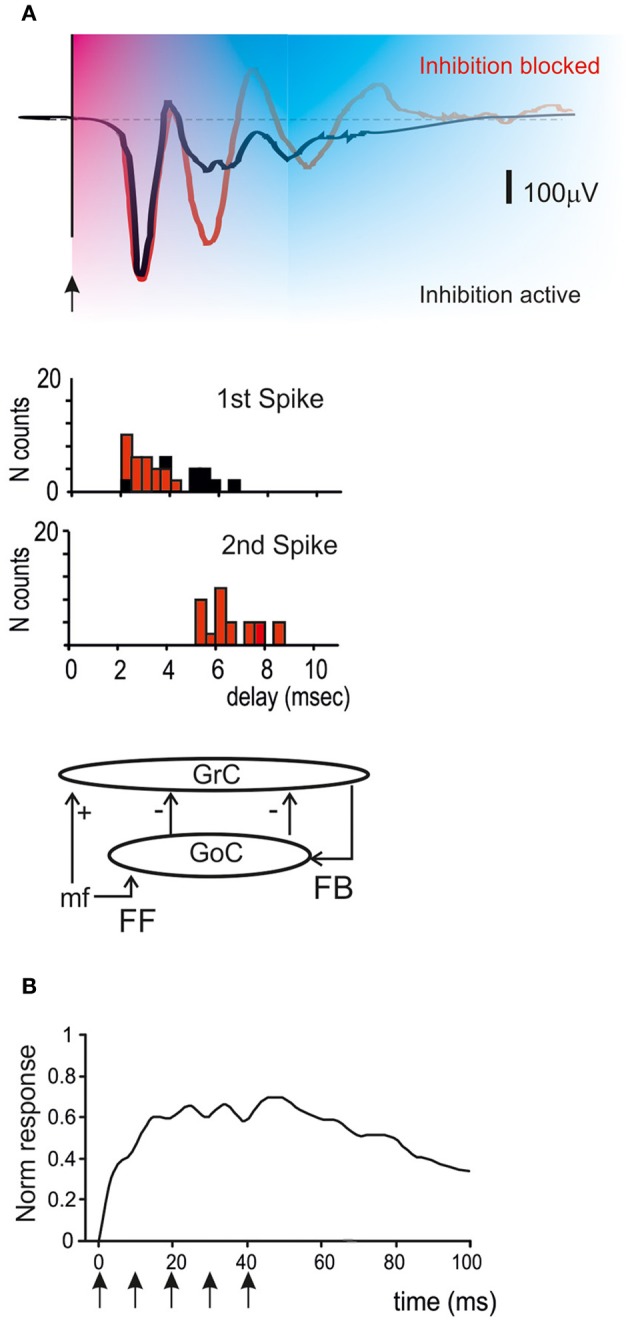
**Golgi cells and the time-window effect. (A)** The time-window effect is generated due to activation of the feedforward and feedback inhibitory loops. After excitation, granule cells are inhibited by the feedforward and feedback loops in sequence leaving a permissive time window of just 4–5 ms. Then inhibition prevails, reducing or blocking the granule cell response. The histograms show spike generation in granule cells in the presence or absence of synaptic inhibition (Mapelli and D'Angelo, 2007; D'Angelo and De Zeeuw, 2009). **(B)** The time-window effect is observed in response to single pulses or short bursts but is suppressed during high-frequency repetitive stimulation of mossy fibers (see text). Stimuli are indicated by arrows [meta-analysis data from Mapelli et al. (2010a)].

**Figure 6:**
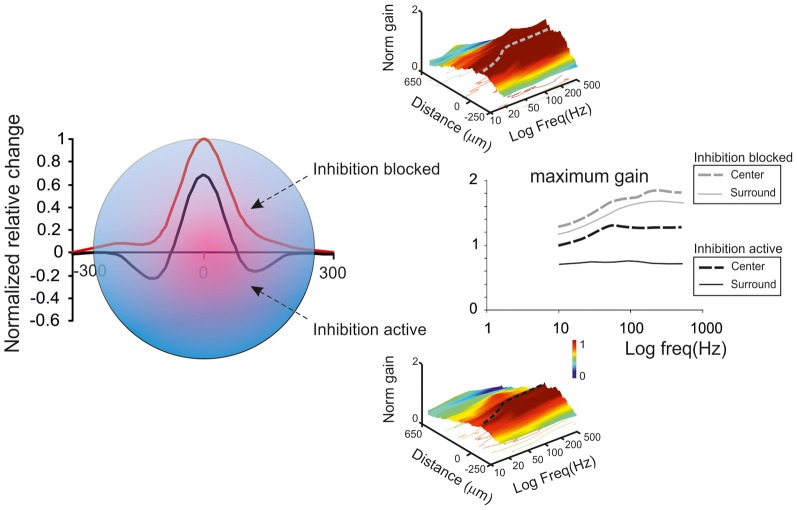
**Golgi cells and center-surround organization.** The center-surround effect is generated by lateral inhibition. After excitation, granule cells in the core are excited much more strongly that those in the surrounding area. This results in the formation of a center-surround structure of about 300 μm diameter. Golgi cells modulate the gain in the center and generate net inhibition in the surround. Golgi cell inhibition tends to decrease the gain and increase the cut-off frequency of signal transmission [meta-analysis data from Mapelli et al. (2010b)].

**Figure 7:**
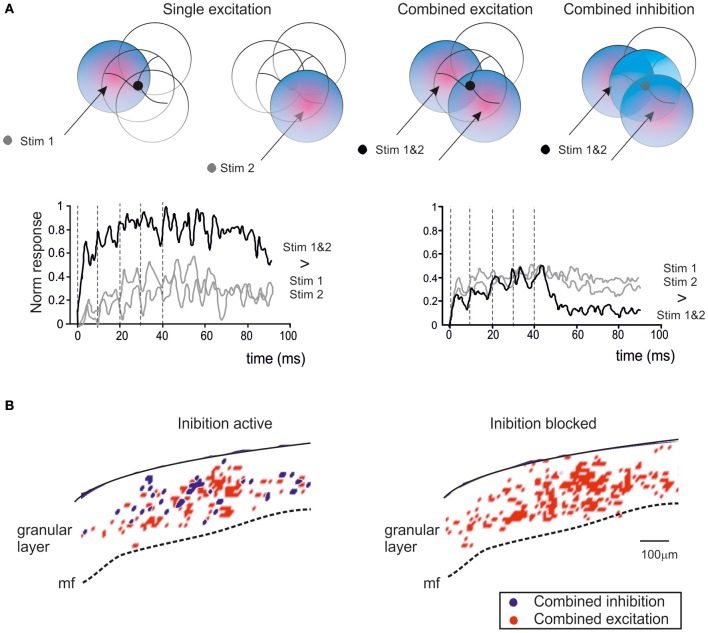
**Golgi cells and combinatorial operations. (A)** When the granular layer is stimulated through multiple mossy fiber bundles (called Stim1 and Stim2), the variable arrangement of synaptic contacts can generate combined excitation of granule cells or combined excitation of Golgi cells. Combined excitation of granule cells determines responses which are stronger than those caused by each individual bundle, while combined excitation of Golgi cells results in granule cell responses smaller than those generated by each individual bundle. The plots show the intensity of granular layer responses (recorded as VSD traces) to a short mossy fiber burst (indicated by broken lines) in areas in which there is either an increase (left: *combined excitation*) or decrease (right: *combined inhibition*) of the response with combined Stim1&2 compared to Stim1 or Stim2 alone (Mapelli et al., 2010a). The effect of combined inhibition becomes evident after stimulation is terminated, probably indicating that during repeated stimulation the inhibitory effect is partially suppressed (see Figure 5). **(B)** Spatial distribution of areas of combined excitation and combined inhibition in the granular layer of a cerebellar slice (data elaborated from VSD recordings). Combined inhibition disappears when Golgi cell transmission is blocked with the GABA-A receptor antagonist gabazine (Mapelli et al., 2010a).

**Figure 8:**
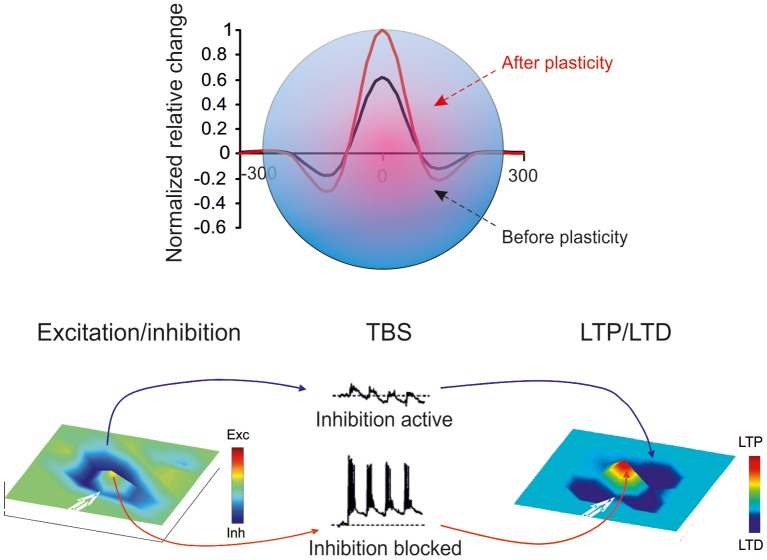
**Golgi cells and long-term synaptic plasticity.** Long-term synaptic plasticity is controlled by Golgi cell synaptic inhibition through regulation of granule cell membrane potential, NMDA channel unblock at the mossy fiber-granule cell synapse, and the subsequent Ca influx in granule cell dendrites (for review see D'Angelo and De Zeeuw, 2009). The result is a marked LTP in the center and LTD in the surround, so that LTP and LTD assume a center-surround organization (Mapelli and D'Angelo, 2007).

Golgi cell inhibition, in addition to regulating the entrainment of granule cells into coherent oscillations, has multiple and complex effects on the way granule cells retransmit signals conveyed by mossy fibers. Mossy fibers usually transmit bursts or long sequences of spikes and granule cells can also emit spike bursts. Thus, once a portion of the granular layer (ideally a “microzone,” see Harvey and Napper, 1991; Mapelli et al., 2010a,b)^1^ is activated by a specific input, both Golgi cells and granule cells are driven by spike bursts. This makes the process of Golgi cell inhibition of granule cells particularly complex, since several non-linear (voltage-dependent, time-dependent and frequency-dependent) effects are called into play. These include the voltage-dependent electroresponsiveness of the Golgi cell, the frequency dependence of synapses impinging on the Golgi cell, and all the specific properties of Golgi cell to granule cell transmission determined by the glomerular organization of the synapses involved (see above). As a whole, burst transmission mechanisms can control the following main operations: the time-window effect (D'Angelo and De Zeeuw, 2009) (Figure 5), the center-surround organization of cut-off and gain of signal transmission (Figure 6) (Mapelli et al., 2010b), the combinatorial rearrangement of granular layer activity (Figure 7), and the sign intensity and extension of long-term synaptic plasticity at the mossy fiber-granule cell relay (Figure 8).

### Spike timing and burst transmission: the time-window effect

Once a burst is conveyed through the mossy fibers, it simultaneously activates the granule cells and the Golgi cells, thus engaging the feedforward inhibitory loop. The effect is to inhibit granule cell firing after an interval corresponding to the sum of transmission and excitation delays along the mossy fiber-Golgi cell-granule cell pathway. The permissive “time window” lasts about 4–5 ms, so that granule cells have time to fire just 1–3 spikes (D'Angelo and De Zeeuw, 2009) (Figure 5). In particular, since mossy fiber-granule cell LTP tends to anticipate granule cell firing and to increase its frequency, while LTD does the opposite (Nieus et al., 2006), long-term synaptic plasticity cooperates with the time-window mechanism in determining the number of spikes emitted by granule cells. This mechanism is likely to have a profound impact on the way bursts are channeled toward the molecular layer and on the way parallel fibers activate Purkinje cells and molecular layer interneurons.

A different regime of inhibition could be set up during prolonged mossy fiber discharges, like those conveyed by tonic units in the proprioceptive (Kase et al., 1980) and vestibular system (Arenz et al., 2008). In this case, the inhibitory action exerted by Golgi cells over granule cells seems to be temporarily suppressed, possibly due to a series of candidate mechanisms including (i) presynaptic reduction of GABA release through tonic activation of GABA-B autoreceptors on Golgi cell synaptic terminals (Mapelli et al., 2009), (ii) presynaptic reduction of GABA release through glomerular crosstalk and activation of mGluRs on Golgi cell synaptic terminals (Mitchell and Silver, 2000a), (iii) post-synaptic reduction of granule cell inhibition through GABA-B receptor-mediated down-regulation of GABA-A IPSCs (Brandalise et al., 2012), (iv) post-synaptic enhancement of granule cell responsiveness by GABA-B receptors reducing an inward rectifier K current in granule cells (Rossi et al., 2006), (v) dendritic activation of Golgi cell mGluR2 enhancing an inward rectifier K current and helping to reduce Golgi cell firing (Watanabe and Nakanishi, 2003). It would be extremely useful to clarify the relevance of these mechanisms during natural circuit activation *in vivo*.

### Regulations of transmission gain and cut-off frequency

In the granular layer, signal transmission along the mossy fiber-granule cell pathway is strongly frequency-dependent (Figure 6), with a high-pass cut off around 50 Hz and a gain which is about two times larger at high compared to low frequencies (Mapelli et al., 2010b). This frequency dependence of gain is regulated by NMDA receptors, which, by exploiting their slow voltage-dependent kinetics and their regenerative voltage-dependent unblock, boost EPSP temporal summation (cf D'Angelo et al., 1995) in the 10–100 Hz range. AMPA receptors, which have kinetic time constants in the millisecond range, allow temporal summation at very high frequencies (200–500 Hz). Thus, the combination of the two receptor-dependent mechanisms allows transmission to be amplified over a broad frequency band covering the natural range of mossy fiber discharge (Chadderton et al., 2004; Jörntell and Ekerot, 2006).

GABA-A receptor activation through the Golgi cell loops, by reducing EPSP temporal summation in granule cells (Armano et al., 2000; for review see D'Angelo, 2008; Kanichay and Silver, 2008; D'Angelo and De Zeeuw, 2009), causes a global transmission decrease over the whole frequency range, which becomes particularly marked at low frequency (Mapelli et al., 2010b). Tonic inhibition could indeed intensify gain reduction at low frequencies (Mitchell and Silver, 2003). Therefore, the granular layer enhances transmission of high-frequency spike bursts through an alternating control of the E/I balance: at high frequencies (>50 Hz) NMDA receptor-dependent depolarization prevails over GABA-A receptor-dependent inhibition, while at low frequencies the opposite occurs. The weakening of the inhibitory loop at high frequencies could also reflect a number of mechanisms mediated by mGluR2 and GABA-B receptors (see above).

### Control of the induction of long-term synaptic plasticity

Long-term synaptic plasticity, in the form of LTP and LTD, is generated by mossy fiber bursts and requires activation of NMDA receptors and calcium influx (D'Angelo et al., 1999; Armano et al., 2000). LTP and LTD induction is bidirectional and follows the Bienenstock-Cooper-Munro theory (Bienenstock et al., 1982), so that the level of calcium discriminates whether LTP or LTD will occur (Gall et al., 2005; Prestori et al., 2013; D'Errico et al., 2009). Since the NMDA receptor-mediated conductance is voltage-dependent, the amount of calcium influx depends critically on the depolarization attained during the induction bursts. Interestingly, the level of granule cell depolarization attained during bursts strictly depends on synaptic inhibition provided by Golgi cells (Armano et al., 2000) and blocking inhibition turns the balance in favor of LTP both *in vitro* (Mapelli and D'Angelo, 2007) and *in vivo* (Roggeri et al., 2008). Considering the E/I balance, high E/I will determine LTP, intermediate E/I will determine LTD, and very low E/I will prevent any plasticity from occurring. Therefore, Golgi cell inhibition is a primary factor in controlling long-term synaptic plasticity in the granular layer circuit (D'Angelo and De Zeeuw, 2009).

## Control of the spatial organization of granular layer activity

A critical aspect of circuit functioning is its topological organization during activity. The granular layer was long thought to effect a *spatiotemporal reconfiguration* of incoming inputs but the physiological basis of this process remained unclear. The classical theoretical models of cerebellar function (Marr, 1969; Albus, 1971) and also subsequent cellular-based computational models (Maex and De Schutter, 1998; Medina and Mauk, 2000) used isotropic connectivity based on cell convergence/divergence ratio statistics, and left the topological problem unaddressed. Nonetheless, recent investigations using VSD imaging and MEA recordings (Mapelli and D'Angelo, 2007; Mapelli et al., 2010a,b) combined with mathematical simulations using realistic computational models (Solinas et al., 2010) have revealed that granular layer activity is topologically organized and that Golgi cells play a central role in determining this organization.

### Center-surround organization

As noted above, a fundamental concept of cerebellar physiology is that a punctate stimulation *in vivo* causes dense activation in granule cell clusters under LTP and LTD control (Roggeri et al., 2008; Diwakar et al., 2009; Ozden et al., 2012). High-resolution analysis *in vitro* showed that areas of dense spiking activity are surrounded by an inhibitory well (Mapelli and D'Angelo, 2007). This center-surround pattern (Figure 6) arises as follows: once a compact bundle of mossy fibers discharges in bursts, a group of granule cells is activated along with local Golgi cells. Since the inhibitory territory of Golgi cells is broader than their excitatory field, and since granule cell excitation diminishes radially from the excitation core, the E/I balance inverts sharply, so that excitation prevails in the core while inhibition prevails in the surrounding area (Mapelli and D'Angelo, 2007). The excited core has a radius of about 30 mm *in vivo* and contains about 260 granule cells with an up to 35% probability of firing; conversely, the firing probability in the surrounding area tends toward zero. This dense-core spiking activity can rise to 50% when Golgi cell inhibition is turned off (Diwakar et al., 2009). Thus, the Golgi cells, by virtue of lateral inhibition, play a critical role in generating center-surround responses, which have three main functional effects: (i) channeling of information through vertical transmission lines, (ii) generation of combinatorial operations among multiple inputs, and (iii) reconfiguration of network topology through control over the induction of long-term synaptic plasticity.

### Signal transmission along vertical channels

A first consequence of granule cell cluster activation is generation of coherent activity in bundles of granule cell ascending axons running vertically toward the molecular layer followed by activation of a group of overlying Purkinje cells (Mapelli et al., 2010b). Along with this, the high E/I balance in the excitation core enhances high-frequency burst transmission, while the prevalence of inhibition in the surround selectively prevents low-frequency transmission (see above) and therefore noise diffusion throughout the network. Therefore, Golgi cells can delimit, focus and sharpen signal transmission through the molecular layer generating vertical transmission channels, as predicted by Bower's investigations (Bower and Woolston, 1983). Enhanced activity-tracking techniques making use of 2PM and VSD imaging could be used to precisely define these signal pathways.

### Generation of combinatorial operations

A second consequence of granule cell cluster activation is that it provides the basis for combining responses generated in neighboring center-surround structures (Mapelli et al., 2010a) (Figure 7). Simultaneous activation of two partially overlapping mossy fiber bundles gives rise to areas of combined excitation and combined inhibition, which are compatible with the concepts of *coincidence detection* and *spatial pattern separation* predicted by theory (Marr, 1969; Albus, 1971; Ito, 1984). Combined excitation appears as an area in which the combination of two inputs is greater than the arithmetic sum of the individual inputs and it is enhanced by GABA-A receptor blockers. Combined inhibition manifests itself as an area where the combination of two inputs results in a reduction of the activity evoked by either one of the two inputs alone and it is prevented by GABA-A receptor blockers. Combinatorial responses occupy small granular layer regions compatible with cluster size and they last for tens of milliseconds. Finally, it should be noted that combined inhibition occurs after bursts are terminated, in keeping with the observation that inhibition is temporarily suppressed during protracted bursts (see above).

The occurrence of combinatorial operations in multiple scattered areas points to specific local circuit topologies. In areas showing combined excitation, mossy fiber convergence onto granule cells needs to prevail (D'Angelo et al., 1995; Jörntell and Ekerot, 2006) over convergence onto Golgi cells, so that Golgi cells can only proportionately reduce granule cell activation. Conversely, in areas showing combined inhibition, the convergence of mossy fibers onto Golgi cells needs to prevail over the convergence onto granule cells, so that Golgi cells can generate effective and strong inhibition during double-bundle stimulation. It is likely that these effects require lateral inhibition (for a general discussion see Buzsaki, 2006), which has indeed been reported in the cerebellum granular layer (Mapelli and D'Angelo, 2007). These combinatorial operations, if engaged by natural input patterns *in vivo*, may be important in order to configure topologically organized spatiotemporal spike sequences to be relayed to Purkinje cells.

As shown in Figure 7, the generation of complex operation is expected to occur at the intersection of *center-surround* structures, so that combined inhibition occurs when most granule cell dendrites are activated in *surround* areas, while combined excitation occurs when most granule cell dendrites are activated in *center* areas. This hypothetical organization, which resembles the overlapping field hypothesis of J.C. Eccles for PCs in the molecular layer (Eccles et al., 1967), awaits for an experimental clarification.

### Reconfiguration of network topology through long-term synaptic plasticity

A third consequence of the center-surround organization of activity derives from the ability of Golgi cell inhibition to control the induction of long-term synaptic plasticity (Figure 8; see also above). The strong excitation in the core favors LTP, while the weak excitation in the surround favors LTD. Thus, the center-surround organization of excitation and inhibition gives rise, after appropriate burst transmission, to a center-surround organization of LTP and LTD. With LTP in the center and LTD in the surround, the topological organization of transmission properties is rendered sharper.

According to Marr (1969), if input trains were to saturate granular layer plasticity, this would interfere with the efficient control of information processing. Experimental evidence (Mapelli and D'Angelo, 2007) supports the tenet that granular layer plasticity is not saturated but, instead, redistributes LTP and LTD in neighboring areas. The circuit therefore maintains a spatially organized homeostatic balance, in which activity is enhanced in certain areas while being reduced in others. Once established, LTP and LTD may be instrumental in regulating the contrast between granular layer fields, extending the original concept of spatial pattern separation (Marr, 1969), in which the excitatory/inhibitory balance of granule cells was predetermined and unchangeable. The LTP and LTD areas may represent channels for differential processing of mossy fiber inputs. In the LTP channel, the delay is reduced and the average frequency of granule cell discharge is enhanced, whereas the opposite is true in the LTD channel (Nieus et al., 2006). Moreover, on the basis of previous data (Mapelli et al., 2010b) and simulations (Solinas et al., 2010) it is expected that LTP channels show an enhanced high frequency transmission gain compared with LTD channels. This prediction awaits experimental confirmation.

## Conclusions

### Evolution of the concepts of granular layer functioning

The original idea of a combinatorial arrangement of connections based on statistics rather than geometry led to the concept that the granular layer activates “sparsely” (i.e., with a very low probability of granule cell firing) and in an isotropic manner. Likewise, the predicted separation of incoming inputs into spatial patterns had no specified topology. Yet, Golgi cells were predicted to play a critical role in these processes (Marr, 1969; Albus, 1971). The granule cell-Golgi cell feedback circuit was then thought to generate temporal dynamics in the system during continuous signal processing (Fujita, 1982). However, no role was envisaged for Golgi cells in controlling long-term synaptic plasticity, simply because the latter was not thought to occur in the granular layer. This view, together with the role attributed to Golgi cells, is now radically changing, as is understanding of granular layer mechanisms as a whole. While basal activity in granule cells is sparse, activity following a localized input becomes concentrated in dense spiking clusters organized in center-surround structures (Diwakar et al., 2011; Ozden et al., 2012). Moreover, LTP and LTD do indeed exist in the granular layer (D'Angelo and De Zeeuw, 2009). Finally, granule and Golgi cells, as well as the synapses in between, show complex temporal dynamic properties (Solinas et al., 2007a,b), which impact on the behavior of the circuit.

### An integrated view of the impact of Golgi cells on the spatiotemporal regulation of granular layer activity

**Figure 9:**
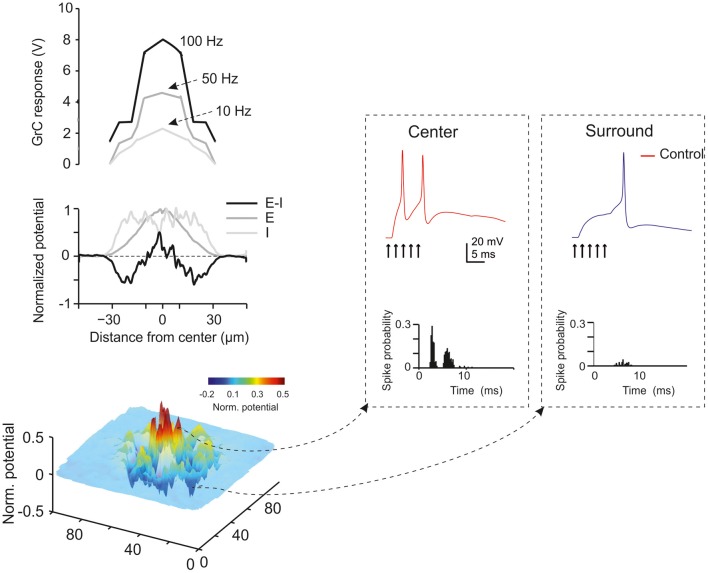
**Golgi cells and granule cell spike coding: modeling predictions.** A large-scale realistic model of the granular layer predicts the impact of Golgi cell synaptic inhibition on granular layer functions including (i) center-surround organization, (ii) time windowing, (iii) gain and cut-off frequency regulation in the center-surround structure (Solinas et al., 2010).

**Figure 10:**
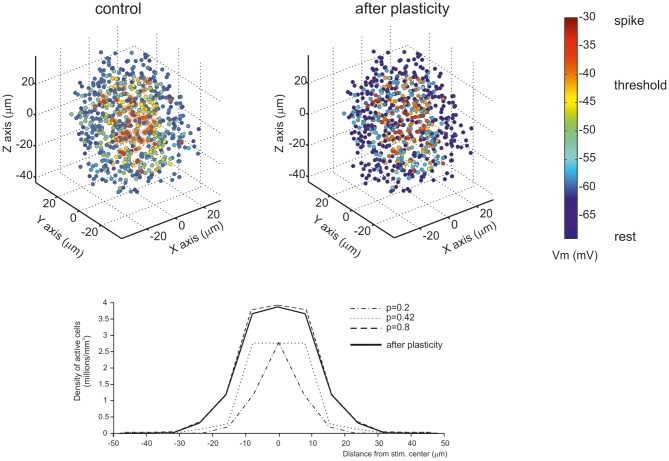
**Golgi cells and granule cell plasticity: modeling predictions.** A large-scale realistic model of the granular layer predicts the impact of LTP and LTD on signal transmission in the center-surround structure. By changing neurotransmitter release probability at the mossy fiber-granule cell synapse (which is itself a function of Golgi cell synaptic inhibition, see Figure 7), the percentage of discharging granule cell increases in the center and decreases in the surround (from D'Angelo and Solinas, 2011).

At present, the main functions of the Golgi cell can be summarized as follows (Figures 9, 10). Golgi cells control the timing and rate of firing inside center-surround activity clusters and sharpen their limits through lateral inhibition. By integrating the activity of multiple center-surround structures, Golgi cells generate different kinds of associative operations. Moreover, Golgi cells regulate the balance between LTP and LTD, concentrating LTP in the more excited and LTD in the less excited areas. By so doing, Golgi cells help to generate selective transmission channels running toward the molecular layer. Interestingly, analysis of granular layer-molecular layer communication suggests that the transmission channels organized by Golgi cells strongly contribute to implementing a vertical organization of the cerebellar cortical function. This might then exploit preferential activation of local Purkinje cells and molecular layer interneurons by the granule cell ascending axon (Bower and Woolston, 1983; Mapelli et al., 2010a,b).

In addition to their effect on the topological organization and plastic rearrangement of activity, Golgi cells make an important contribution to the control of granular layer temporal dynamics. By sustaining low-frequency oscillations generated by randomly distributed inputs through their feedback loop, Golgi cells allow temporal binding of granule cell activity. By exploiting phase-resetting mechanisms through the feedforward loop, Golgi cells can convey specific mossy fiber signals through the network. In this operating mode, Golgi cells can control the number of spikes and the duration of granular layer activity following an impulse, implementing a “time-window” control mechanism, which operates differentially in the center and in the surround and is modulated by LTP and LTD.

All in all, Golgi cells seem to be the fundamental elements coordinating the spatiotemporal transformation of spike patterns occurring at the cerebellum input stage. This activity is deeply interrelated with that occurring in the cerebral cortex (Ros et al., 2009), which means that it would probably be very useful to understand how Golgi cells are activated in relation to the coordinated activity taking place in cerebrocortical loops during specific sensorimotor or cognitive operations.

Contrast enhancement in the granular layer and Purkinje cell selection could contribute to the spatiotemporal recoding of mossy fiber information predicted by theoretical network analysis (Eccles, 1973; Pellionisz and Llinás, 1979; Pellionisz and Llinas, 1980, 1982; Medina and Mauk, 2000; De Schutter and Bjaalie, 2001; Llinas and Roy, 2009) and could play a role in cerebellar receptive field reshaping after sensory stimulation (Jörntell and Ekerot, 2006). Artificial network models indicate that combining lateral inhibition with Hebbian learning regulates competition between neighboring areas causing the emergence of self-organized topology, feature abstraction, and generalization (Kohonen, 1984; Rieke et al., 1997). It is important to note in this respect that, by implementing operations of the AND and XOR category, Golgi cells may constitute a *hidden layer* within the granular layer circuit, thus reinforcing the ability of the cerebellar input stage to perform extensive pattern recognition, categorization and generalization of mossy fiber inputs (Spitzer, 1998). New imaging and MEA techniques as well as appropriate large-scale network simulations may help to shed light on the potential occurrence of these properties in the granular layer of the cerebellum.

### Conflict of interest statement

The authors declare that the research was conducted in the absence of any commercial or financial relationships that could be construed as a potential conflict of interest.

## Footnotes

^1^Transient Na current (I_Na − t_); persistent Na current (I_Na − p_); resurgent Na current (I_Na − r_); high-voltage-activated Ca current (I_Ca − HVA_); Ca-dependent K current of the BK-type (I_K − BK_); Ca-dependent K current of the SK-type (I_K − AHP_); delayed-rectifier K current (I_KV_); slow K current of the M-type (I_K − slow_); fast-inactivating K current of the A-type (I_K − A_); slow inward-rectifier H-current (I_h_).

^2^The cerebellum is composed of several hundred or thousand microzones or microcompartments, which are thought to represent effective cerebellar functional units [for a recent review see D'Angelo and Casali (2012)]. These have a complex relationships with stripes, zones and multizonal microcomplexes, which are also though to represent effective functional modules (Apps and Hawkes, 2009). A module is a conglomerate of several, non-adjacent parasagittal bands of Purkinje cells projecting to specific areas of deep cerebellar nuclei and gating segregated projections from the inferior olive (Oberdick and Sillitoe, 2011). Likewise, the mossy fibers projecting to a certain group of Purkinje cells through the granular layer also project to the deep cerebellar nucleus neuron receiving input from those Purkinje cells (Voogd et al., 2003).
